# After-School Based Obesity Prevention Interventions: A Comprehensive Review of the Literature

**DOI:** 10.3390/ijerph9041438

**Published:** 2012-04-16

**Authors:** Paul Branscum, Manoj Sharma

**Affiliations:** 1 Department of Health & Exercise Science, The University of Oklahoma, 1401 Asp Avenue, HHC 112, Norman, OK 73019, USA; 2 Health Promotion and Education, The University of Cincinnati, P.O. Box 210068, Cincinnati, OH 45221, USA; Email: sharmamj@ucmail.uc.edu

**Keywords:** child obesity, literature review, after school

## Abstract

The purpose of this article was to review primary prevention interventions targeting childhood obesity implemented in the after school environment from 2006 and 2011. A total of 20 interventions were found from 25 studies. Children in the interventions ranged from kindergarten to middle schoolers, however a majority was in the 4th and 5th grades. Most of the interventions targeted both physical activity and dietary behaviors. Among those that focused on only one dimension, physical activity was targeted more than diet. The duration of the interventions greatly varied, but many were short-term or brief. Many interventions were also based on some behavioral theory, with social cognitive theory as the most widely used. Most of the interventions focused on short-term changes, and rarely did any perform a follow-up evaluation. A major limitation among after school interventions was an inadequate use of process evaluations. Overall, interventions resulted in modest changes in behaviors and behavioral antecedents, and results were mixed and generally unfavorable with regards to indicators of obesity. Recommendations for enhancing the effectiveness of after school based childhood obesity interventions are presented.

## 1. Introduction

Obesity is a major public health concern in today’s society. This is especially true with regards to children, given that obesity has tripled in this group and is a major risk factor for obesity in adulthood [1]. According to the most recent National Health and Nutrition Examination Survey (NHANES) completed in 2007−2008, 31.7% of children between the ages of 2 to 19 years were overweight (≥85th percentile), 16.9% were obese (≥95th percentile), and 11.9% were severely obese (≥97th percentile) [2]. Of concern, obesity has been associated with numerous metabolic and psychological conditions, which now occurs much earlier in life compared with previous generations [3,4,5,6]. 

Health promoting interventions implemented earlier in life, targeting modifiable risk factors such as diet and exercise, are likely to have a positive contribution to the prevention of child and adult onset obesity. To have the greatest impact, public health interventions should occur in venues that service large and accessible segments of the target population. For children, schools are an obvious venue of choice. Schools provide a captive audience of children and adolescents between the ages of 5−18 years. Many schools are also equipped with resources that can aid in the facilitation of health behavior change, such as gymnasiums and green spaces that provide a safe environment for physical activity, and trained health professionals, such as physical education teachers, health teachers, and school nurses who can organize and implement formal and informal health programs. To date many obesity prevention interventions have been implemented in the school setting, however recent findings have not been overwhelmingly supportive to their effects. Meta-analyses conducted by Katz and colleagues [7], Gonzalez-Suarez and colleagues [8], Kanekar and Sharma [9], and Cook-Cottone and colleagues [10] all conclude that the changes on BMI as a result of these strategies are generally small or statistically insignificant. 

While more research is needed in this area, the issue of accessibility, or the ability researchers have to reach children in a selected venue, has become increasingly important in recent years. With a greater focus on standardized testing, schools and school districts are now under increased pressure to focus efforts on testable academic areas, which often excludes health and physical education [11]. It is reasonable to expect that an atmosphere conducive to health can help to improve learning outcomes however. Results from a recent study showed a significant positive relationship between physical fitness and math and English achievement tests scores among 4th and 5th grade children [12]. Nonetheless, many schools have decided that time can no longer be devoted to these areas, and some are greatly reducing or all together eliminating opportunities for health and physical education from their curriculums [13]. Nationwide, only 4.2% of elementary schools require daily physical education for all students [14]. Even recess is no longer a required or implemented in all schools; currently 61.5% of school districts require or recommend elementary school recess for an appropriate amount of time [14]. 

With larger demands placed upon schools, it is vital that researchers and practitioners target alternative venues that still service a large amount of children but are also accessible. One potential and promising setting is through after school programs (ASP’s). From 1985 to 1998 the percentage of children (6−17) with both parents in the work force increased from 63% to 71%, making a larger demand for both before and after school programming [15]. Currently 8.4 million youth (K-12th grade) participate in some form of ASP [16]. ASP’s aim to provide a safe and structured environment for children during the hours immediately following the end of the school day, and oftentimes have the ability to offer the same opportunities schools have to facilitate health promotion and aid in the prevention of child and adolescent obesity. For example, many ASP’s are implemented in the school environment itself, giving the program access to the gymnasium and outdoor spaces for structured or unstructured physical activity. Physical activities are also commonplace in ASP’s, which is ideal since children often need an outlet to relax after attending a long day of school [17]. ASP’s can also impact dietary habits and preferences of children. Children left at home unsupervised have a greater opportunity to engage in unhealthy eating habits such as snacking on high calorie foods, while children in ASP’s commonly have designated snack times, which have the ability to limit the types and portion sizes of snacks that precede dinner time. ASP’s also have the opportunity to encourage healthy snacking behavior by providing repeated exposures to important food groups such as fruits, vegetables and low or nonfat diary products. This setting is also advantageous because unlike schools, ASP’s often encourage and search for outside activities that are either not offered during the school day or can complement school subject matter, including sports, arts and drama, cultural enrichment, science and health education. Positive outcomes have also been associated with attending ASP’s for children, such as greater academic achievement, lowered behavioral problems and increased social competence [17]. 

Compared to school-based interventions, less work has been done implementing and evaluating after school based obesity prevention interventions. There has been recent interest in this area however, and as our knowledge base grows it is important to review the existing literature in order to describe the current state of research and practice and make recommendations for future researchers. Therefore, the purpose of this study was to review obesity prevention programs implemented during the after school time frame in the United States. 

## 2. Methods

**Figure 1:**
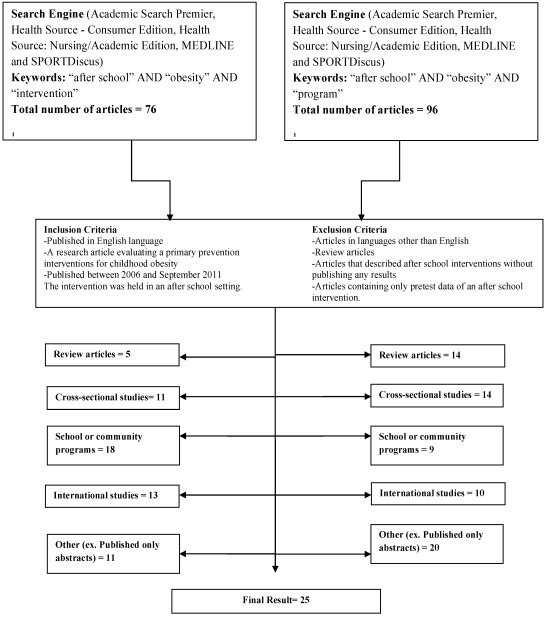
Article selection process to find articles for this review.

An extensive literature search was conducted to collect studies for inclusion in this review. Two separate searches were conducted by both authors of this study using the databases Academic Search Premier, Health Source—Consumer Edition, Health Source: Nursing/Academic Edition, MEDLINE and SPORTDiscus. The first search used the keywords “after school”, “obesity” and “intervention” and yielded 76 abstracts, and the second search used keywords “after school”, “obesity” and “program” and yielded 96 abstracts. Inclusion criteria for studies in this review were: (1) publication in English language; (2) a research article evaluating a primary prevention interventions for childhood obesity (or an intervention aiming to prevent obesity rather than treat obesity); (3) publications between 2006 and September 2011 and (4) the intervention was held in an after school setting. Exclusion criteria were articles in languages other than English, review articles, articles that described after school interventions without publishing any results, and articles containing only pretest data of an after school intervention. Both authors read and reviewed abstracts from these searches, and using the inclusion and exclusion criteria, retrieved articles. After reading the articles, both of the authors decided which to include. This is further illustrated in Figure 1. Important elements of each study that will be reviewed include: the name of the intervention, the theory utilized, the duration of the intervention and a brief description of the program, original reference, design and sample, and salient findings.

## 3. Results

A total of twenty-five studies, evaluating twenty interventions from 2006 to 2011 met the inclusion criteria for this study, and are presented in Table 1. Articles are reviewed in chronological order, with the earliest studies reviewed first. 

Table 2 shows important elements of each study design. The first element was the presence and description of three types of impact measures including antecedents of behavior (ex. self-efficacy), behaviors (ex. diet or physical activity), and measures of weight status, body composition or some other functional assessment such as aerobic fitness or blood pressure. Other elements that were reviewed include the presence of an *a priori *sample size calculation, whether some type process evaluation was completed and reported, the number of measurements reported, and the setting of the after school program. A discussion of these elements will follow.

**Table 1:** Summary of primary prevention interventions targeting obesity prevention in the after school setting.

#	Intervention	Theory	Intervention Duration & Description	Study	Design & sample	Salient findings
1.	*Youth Fit For Life*	Social Cognitive Theory	-Delivered in three 45-minute sessions over 12 weeks. -Program consisted of 4 components, targeting: (1) Cardiovascular exercise by noncompetitive games (2) Resistance exercise using resistance bands (3) Nutrition/health information (4) Behavioral skills training	Annesi, 2006 [18]	Quasi-Experimental TX: *n* = 84 (2005) TX: *n* = 41 (2003) CNT: *n* = 40 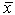 x age = 10.8	-Significant improvements for PA (*p* < 0.001), physical self-concept (*p* < 0.013) and self-efficacy for exercise barriers (*p* < 0.001) in the treatment group and no changes in the control.
				Annesi, Faigenbaum, *et al*. 2008 [19]	Quasi-Experimental TX: *n* = 146 CNT: *n* = 123 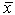 age = 10.6	-Significant improvements for all self-appraisal factors (ex. general self (*p* < 0.003)), mood factors (ex. Tension (*p* < 0.001)) and PA (*p* < 0.001) within the treatment group. -Measures also significantly different between the TX and CNT groups at the time of posttest (*p* < 0.001).
				Annesi, Moore, *et al*. 2008 [20]	Quasi-Experimental TX: *n* = 217 Overall 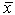 age = 9.7	-Significant improvement for PA (*p* < 0.001) from baseline to the end of the program. -Significant negative predictors of PA post-intervention included frequency of PA at baseline (*p* < 0.001) and age (*p* < 0.007)
				Annesi, *et al*. 2009 [21]	Pilot Study Total *n* = 43 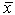 age = 9.0 years	-Significant improvements found for BMI (*p* < 0.03), strength (*p* < 0.001), endurance (*p* < 0.04), engagement in PA (*p* < 0.01), vegetable intake (*p* < 0.02), self-efficacy for PA (*p* < 0.002) and physical self-concept (*p* < 0.05), but not flexibility, or fruit intake.
				Annesi, *et al*. 2011 [22]	Quasi-Experimental TX :*n* = 121 TX plus HopSports® video-system: *n* = 171 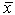 age = 7.3 years	-Significant improvements for BMI-percentile (*p* < 0.001), muscular strength (*p* < 0.001), and cardio-respiratory endurance (*p* < 0.007) within both groups, but no difference between groups.
2.	*Nutrition & Media Intervention*	Social Cognitive Theory	-Delivered in 12 two-hour sessions over 6 weeks. -Program included education for nutrition, media literacy and health communication.	Evans, *et al*. 2006 [23]	Pilot Study TX: *n* = 18 CNT: *n* = 21 4th and 5th grade children	-Significant improvements for motivation (*p* < 0.013), home nutrition environment (*p* < 0.02), and perceived parental support (*p* < 0.04), but not fruit/vegetable intake, self-support or self-efficacy.
3.	*Pilates Program*	None stated	-Delivered every day for 4 weeks. -Program included basic training in Pilates.	Jago, *et al*. 2006 [24]	Pilot Study TX: *n* = 16 CNT: *n* = 14 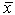 age = 11.2 years	-Significant improvements for BMI-percentile (*p* < 0.039), but no other measure (ex. blood pressure)
4.	*10-Month Physical Activity Intervention*	None stated	-Delivered everyday school day for 110 minutes for a total of 10-months -For each session: 30 min. for homework; 25 min. PA skills development; 35 min. PA; and 20 min. toning/stretching	Barbeau, *et al*. 2007 [25]	RCT TX: *n* = 118 CNT: *n* = 83 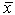 age = 9.5 years	-Average attendance was 54% (2.5 days/week). -Significant improvements for BMI-percentile (*p* < 0.008), body composition (*p* < 0.0001), cardiovascular fitness (*p* < 0.047) and overall PA (*p* < 0.0006) were found for the treatment group, compared with the control group.
				Howe, *et al*. 2011 [26]	RCT TX: *n* = 62 CNT: *n* = 44 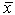 age = ~9.8 years	-Average attendance was 57.7% (2.5 days/week). -Significant improvements for children attending ≥60% of intervention for MVPA (*p* < 0.04), BMI (*p* < 0.0034), and body composition (*p* < 0.019) compared to the control group.
5.	*Kids Living Fit*	Social Cognitive Theory	-Delivered once per week for 12 weeks. -Sessions included various exercise and dietary components	Speroni, *et al*. 2007 [27]	Quasi-Experimental TX: *n* = 80 CNT: *n* = 105 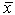 age ≈ 9.3 years	-Average attendance was 82%. -Children in TX group experienced a significant decrease in BMI-% (*p* < 0.01), while those in CNT group had a significant increase (*p* < 0.01).
6.	*Georgia Fitkid*	None stated	-Delivered for 2 hours every school day for three-years. -For each session, 40 min. for eating a healthy snack and 80 min. for PA.	Gutin, *et al*. 2008 [28]	RCT TX: *n* = 148 CNT: *n* = 168 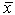 age = 8.5 years	-Significant improvements for bone density (*p* < 0.01), fat-free soft tissue (*p* < 0.01), weight (p < 0.01), height (*p* < 0.01), and body mass index (*p* < 0.05) were found for TX group. -Also a group x time interaction for fitness (*p* < 0.01) and body fat % (*p* < 0.05) but benefits were not sustained during the summer.
7.	*Be a Fit Kid*	Precede/ Proceed model	Delivered 3 times each week in 2 hours sessions for 12 weeks. -Program consisted of a PA, diet and parent component	Slawta, *et al*. 2008 [29]	Pilot Study TX: *n* = 75 6 to 12 years	-Significant improvements for body composition (*p* < 0.001), fitness (*p* < 0.001), nutrition knowledge (*p* < 0.001), some dietary habits (ex. Total fat intake (*p* < 0.001), and for those who participated in at least 75% of the program, near significant reductions in total cholesterol (*p* < 0.059) and triglyceride levels (*p* < 0.099).
8.	*Food Fit*	Social Cognitive Theory	-Delivered six 45-minute sessions over 6 weeks. -Program consisted of skills training to identify healthier foods.	Branscum, *et al*. 2009 [30]	Pilot Study TX: *n* = 58 3rd, 4th and 5th grade children	-Significant improvements for overall dietary behaviors (*p* < 0.001), and behavior antecedents for some lessons.
9.	*Club Possible*	Social Cognitive Theory	-Implementation varied by site. -Program consisted of education and behavior change activities for increasing PA, and improving healthy eating.	Huberty, *et al*. 2009 [31]	Quasi-Experimental TX: *n* = 670 Age range: 5 to 11	-BMI-percentile significantly decreased among children (*p* < 0.0001). -There were no changes in PA self-efficacy, or social support among children ages 7−9 or 10−12, and only 7−9 age group significantly increased PA enjoyment (*p* < 0.002).
10.	*SCORES*	None stated	-Delivered three 60-minute sessions weekly for 18 weeks. -A program that uses soccer to teach literacy in low-income areas.	Madsen, *et al*. 2009 [32]	Pilot Study TX: *n* = 178 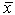 age = 9.7 years	Overall physical fitness scores significantly increased (*p* < 0.001). No significant change was reported for overall BMI-percentile, except there was a significant decrease among Asian children (*p* < 0.001).
11.	*Ready. Set. ACTION!*	Social Cognitive Theory	-Delivered fourteen 2-hour sessions and eight weekly booster session. -Incorporated theater activities with health promotion activities	Neumark-Sztainer, *et al*. 2009 [33]	Pilot Study TX: *n* = 51 CNT: *n* = 45 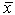 age = 10.3 years	-No significant differences in changes for BMI-%, diet, PA, family/home environment or SCT constructs (except for self-efficacy for PA (*p* < 0.028))
12.	*Tommie Smith Youth Athletic Initiative (TSYAI) *	Trans-theoretical Model	-Delivered three 90-minute sessions/week for 14-weeks. -Included track & field and other PA games with various health promotion activities.	Topp, *et al*. 2009 [34]	Pilot Study TX: *n* = 63 K-5th grade children	-Overall significant improvement for cardiovascular fitness (*p* < 0.01), but no change for BMI-% and percentage body fat. -Children also consumed significantly more green vegetables (*p* < 0.02) and less fruit juice (*p* < 0.02), but there were no other changes in diet.
13.	*HOP’N*	Social Cognitive Theory	-Delivered over three years. -Contained daily PA (30-min.), healthy snacks, and weekly nutrition or PA educational experience.	Dzewaltoski, *et al*. 2010 [35]	RCT Tx: *n* = 134 Cnt: *n* = 112 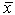 age = ~ 9.2 years	-No changes in BMI z-score observed. -Significant improvements found PA (*p* < 0.04) and sedentary behaviors (*p* < 0.01), especially among overweight/obese children.
14.	*Smart Snack*	Social Cognitive Theory	-Program included three-90 min workshops implemented weekly. -Various program activities (ex. games) implemented to teach children healthy eating habits.	Freedman, *et al*. 2010 [36]	Pilot Study TX: *n* = 63 Age range: 9−14 years	-Of various dietary measures milk (*p* < 0.05), vegetables (*p* < 0.05), and water intake (*p* < 0.05) significantly increased at posttest, but only water (*p* < 0.01) remained significant at the 3-month follow-up.
15.	*NutriActive*	None stated	-Delivered everyday for 90-min, for 4-weeks. -Program included PA, snack and supervised non-structured play.	Matvienko, *et al*. 2010 [37]	Quasi-Experimental TX: *n* = 42 CNT: *n* = 28 K and 1st grade children	-Significant improvements for some fitness (ex. Push-ups (*p* < 0.001)) and all motor skill tests at 4 weeks (*p* < 0.001), however at the 4-month follow-up these improvements were no longer different between the TX and CNT group.
16.	*GEMS (Girls’ health Enrichment Multi-site Studies)*	Social Cognitive Theory	-Delivered everyday for two hours, for 2-years. -Program consisted of teaching traditional and current dance, and strategies for reducing screen time.	Robinson, *et al*. 2010 [38]	RCT TX: *n* = 134 CNT: *n* = 127 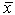 age = 9.4 years	-No change for BMI in TX group, but reported significant improvements in cholesterol (*p* < 0.001), LDL (*p* < 0.001), and depressive symptoms (*p* < 0.02).
17.	*SNAP (Scouting Nutrition & Activity Program)*	Social Cognitive Theory	-Delivered eight 60-90 minute sessions over four months. -Program consisted of: (1) An educational curriculum delivered by troop leaders; (2) Troop meeting policies; and (3) Badge assignments completed at home.	Rosenkranz, *et al*. 2010 [39]	RCT TX: *n* = 33 CNT: *n* = 39 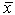 age = 10.6 years	-Intervention troops significantly increased PA (*p* < 0.001) but no change for control troops. -No significant intervention effect on girl’s BMI z-scores, PA, fruit and vegetable consumption, or SSB consumption. -No significant intervention effect for parents FV consumption, PA, or SSB consumption.
18.	*Food and Fitness Fun Education Program (FFFEP)*	None stated	-Delivered weekly for 30−60 minute sessions over sixteen weeks. -Program included lessons on healthy eating and physical activity and daily physical activities were implemented.	Carson, *et al*. 2011 [40]	Quasi-Experimental TX: *n* = 1810 K-5th grade children	-Significant improvements in nutrition (*p* < 0.01) and PA knowledge (*p* < 0.01) for TX group. -Parent surveys suggested that their child and own diet and PA behaviors changes as a result of the program.
19.	*LA Sprouts*	None stated	-Delivered weekly for 90 minute sessions over twelve weeks. -Program included gardening, cooking and nutrition education.	Davis, *et al*. 2011 [41]	Quasi-Experimental TX: *n* = 34 CNT: *n* = 70 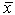 age = ~ 9.8 years	-Significant improvements for fiber (*p* < 0.04) and diastolic blood pressure (*p* < 0.04) for TX group, compared to CNT group. -For the overweight/obese sample, TX group significantly decreased BMI (*p* < 0.04) compared with CNT group.
20.	*Bienestar & CATCH*	Social Cognitive Theory	-Delivered twice weekly for 65−90 minute sessions over twelve weeks. -Program included a bi-lingual health education program, and the PA component of CATCH.	de Heer, *et al*. 2011 [42]	RCT TX: *n* = 242 CNT: *n* = 326 Spillover: *n* = 236 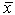 age = 9.2 years	-Significant improvements for BMI-% (*p* < 0.045), aerobic capacity (*p* < 0.012) and intentions to eat healthy (*p* < 0.046) found for ASP’s that reported higher intervention exposure.

**Table 2:** Important research elements of primary prevention interventions targeting obesity prevention in the after school setting.

#	Study	Outcome Measures			A priori Sample Size Calculation	Process Evaluation	Number of Measurements	Setting
		Antecedents of behavior	Behaviors	Body Composition or Other Functional Outcome				
1.	Annesi, 2006 [18]	Physical self-concept, and self-efficacy for exercise barriers	PA	None	No	Yes	Two (pre & post)	YMCA ASP
	Annesi, Faigenbaum, *et al.* 2008 [19]	Four self-appraisal (ex. general self) and two mood variables (ex, tension)	PA	None	Yes	No	Two (pre & post)	YMCA ASP
	Annesi, Moore, *et al.* 2008 [20]	None	PA	BMI-%	Yes	No	Two (pre & post)	YMCA ASP
	Annesi, *et al.* 2009 [21]	Self-efficacy for PA, physical self-concept, and general self	PA and FV intake	BMI, muscular strength, cardio-respiratory endurance, and flexibility	No	Yes	Two (pre & post)	YMCA ASP
	Annesi, *et al.* 2011 [22]	None	None	BMI-%, muscular strength, and cardio-respiratory endurance	Yes	Yes	Two (pre & post)	YMCA ASP
2.	Evans, *et al.* 2006 [23]	Home nutrition environment, self-efficacy, motivation, social support, and perceived parental support	FV intake	None	No	Yes	Two (pre & post)	School Affiliated ASP
3.	Jago, *et al.* 2006 [24]	Perceived exertion and enjoyment.	None	BMI-%, waist circumference, and blood pressure.	No	Yes	Two (pre & post)	YMCA ASP
4	Barbeau, *et al.* 2007 [25]	None	PA	BMI-%, waist circumference, body composition and cardiovascular fitness	No	Yes	Two (pre & post)	School Affiliated ASP
	Howe, *et al.* 2011 [26]	None	PA	BMI-%, waist circumference, body composition and cardiovascular fitness	No	Yes	Two (pre & post)	School Affiliated ASP
5.	Speroni, *et al.* 2007 [27]	Body self-perception, and satisfaction for favorite foods and activities.	None	BMI-%, and waist circumference	No	Yes	Three (pre, post and 3-month follow-up)	School affiliated ASP
6.	Gutin, *et al.* 2008 [28]	None	None	Body composition, and aerobic fitness	No	Yes	Six (pre and post each year for three years)	School affiliated ASP
7.	Slawta, *et al.* 2008 [29]	Diet knowledge	Diet	Fitness, BMI, body composition, lipids and lipoproteins.	No	Yes	Two (pre & post)	School affiliated ASP
8.	Branscum, *et al.* 2009 [30]	Self-efficacy, outcome expectancies, and behavioral capabilities	Diet	None	No	Yes * reported elsewhere	Two (pre & post)	School affiliated ASP
9.	Huberty, *et al.* 2009 [31]	Enjoyment, self-­efficacy, and social support.	None	BMI	No	Yes	Two (pre & post)	Various ASP’s
10.	Madsen, *et al.* 2009 [32]	None	None	Fitness and BMI	No	No	Two (pre & post)	School affiliated ASP
11.	Neumark-Sztainer, *et al.* 2009 [33]	Self-efficacy, enjoyment for PA & FV, weight concerns, body satisfaction, self-worth and home environment.	Diet, PA, TV viewing, and response to satiety cues	BMI/BMI z-score	No	Yes	Two (pre & post)	School affiliated ASP
12.	Topp, *et al.* 2009 [34]	None	Diet	Cardiovascular fitness, BMI-%, waist circumference and body composition.	No	Yes	Two (pre & post)	School affiliated ASP
13.	Dzewaltoski, *et al.* 2010 [35]	None	PA and sedentary activities	BMI z-score	Yes	Yes	Six (beginning, mid, and end of year for two years)	School affiliated ASP
14.	Freedman, *et al.* 2010 [36]	None	Diet	None	No	Yes	Three (pre, post and 3-month follow-up)	Library ASP
15.	Matvienko, *et al.* 2010 [37]	None	None	BMI, waist circumference, and motor skills	No	No	Three (pre, post and 4-month follow-up)	School affiliated ASP
16.	Robinson, *et al.*2010 [38]	PA preference, over concern with weight, body size perception, depressive symptoms, self-esteem, and school performance.	PA, screen time, eating meals while watching TV, and diet.	BMI, waist circumference, body composition, blood pressure, heart rate, fasting lipids, glucose and insulin.	Yes	Yes	Six (beginning, mid, and end of year for two years)	Community center ASP
17.	Rosenkranz, et al. 2010 [39]	None	PA, and diet	BMI z-score,	Yes	Yes	Two (pre & post)	Girl Scout ASP
18.	Carson, et al. 2011 [40]	Nutrition and PA knowledge,	PA, and diet (parent survey)	None	No	No	Two (pre & post)	School affiliated ASP
19.	Davis, et al. 2011 [41]	None	Diet	BMI-%, body composition, waist circumference, and blood pressure	No	Yes	Two (pre & post)	Community center ASP
20.	de Heer, et al. 2011 [42]	Diet intentions and knowledge,	None	BMI-%, aerobic capacity	Yes	No	Two (pre & follow up)	School affiliated ASP

* Abbreviations (ASP = after school program; PA = physical activity; apo B = apolipoprotein B; BMI-% = body mass index percentile; BMI = body mass index; FV = fruit and vegetables)

## 4. Discussion and Conclusions

The purpose of this study was to evaluate current primary prevention interventions implemented in the after school setting for child and adolescent obesity. Based on this review it is evident that the after school time frame is increasing in popularity for intervention and research. The experimental rigor of the studies reviewed in this article greatly varied as approximately one third (*n* = 7 studies) were RCT’s, a third were quasi-experimental studies (*n* = 9 studies) and a third were pilot studies (*n* = 9 studies). It is clear more RCT’s are needed in this area, since they are generally considered the gold standard for program evaluation. A greater number of RCT’s will also be useful for conducting more in-depth reviews in the future, such as a meta-analysis to yield a common effect size for measures such as BMI-percentile, and behaviors such as physical activity. 

Obesity prevention programs were also incorporated into many extracurricular activities that attracted children to participate. For example, one intervention utilized Girl Scout troops, which is nationally known as an enrichment program for young girls. During the program troop leaders served as positive role models and merit badges were given to incentivize the young girls to adopt healthy behaviors [38]. Sports that some children may not have experience with were also used to promote physical activity, including Pilates [23], soccer (for inner city youth) [31], and culturally tailored dance routines [37]. Communications was utilized in an intervention to help children learned aspects of media campaigning, in which they developed refrigerator magnets, a web site, a commercial, and a rap song to promote healthy behaviors for among their family members [22]. Other innovative programs included teaching various aspects of theater production, which culminated with a play performances at the school [32], and teaching children agriculture through developing and maintaining a community gardening [40]. From these examples it is clear that the opportunities in the after school environment are vast. Researchers should use this opportunity to incorporate obesity prevention strategies in fun and exciting activities that are available to them, and that also peak the interest of their children. 

The age and/or school grade range of the children in the studies in this review were from kindergarten through middle school, however the average age range was from 9 to 10 years. This indicates that children were generally in the fourth or fifth grade. Targeting this age group is useful since dietary and physical activity behaviors start to develop in these years and interventions designed to influence and build healthy behaviors at this juncture have the potential for long-term impact. This might also be indicative of the age-range researchers and practitioners should expect to find in this setting. As children grow older parents may be more likely to allow their children to stay home unsupervised, and when they enter middle school (the sixth or seventh grade) after school programs are likely replaced by sports or academic teams. Therefore, this may be a limitation of this setting; while accessibility is high, the availability of older children including preteens and teens, is likely much lower. More research is needed to address this issue.

A little over half of the interventions in this review targeted both nutrition and physical activity behaviors (*n* = 12), while four aimed to modify physical activity alone, and three aimed to modify nutrition behaviors alone. Among the intervention that targeted nutrition behaviors either alone or with physical activity, fruit and vegetable consumption and snacking were the two most common behaviors targeted. The pattern that a majority of interventions targeted both physical activity and nutrition behaviors is similar to that of school-based obesity prevention interventions [43]. While multifaceted, comprehensive programs are beneficial and ultimately needed for obesity prevention there is however some value in testing single-component programs to better test their efficacy. Therefore, we recommend more studies are needed for testing both types of interventions: the effectiveness of multi-component interventions and the efficacy of single-component interventions. Results from efficacy trials should also ultimately inform researchers of efficacious practices that can be used in multi-component interventions. 

Another finding was that a majority (*n* = 13) of the interventions reviewed were based on some behavioral theory, a trend that is similar to school-based obesity prevention interventions [43]. Theories are beneficial for promoting healthy behaviors for several reasons; for example they discern measurable intervention objectives, and provide guidance for intervention strategies. Social cognitive theory (SCT) was the most commonly used theory among the interventions in this review, which posits that human behavior can be explained by reciprocal determinism, or a continuous interaction between behavioral, personal and environmental factors [44]. This was not surprising, given the popularity of this theory in obesity prevention research. In a meta-analysis spanning from 1985 to 2003 authors reviewed randomized controlled trials (RCT’s) designed to favorably impact nutrition and physical activity among children and interventions that were most successful were implicitly or explicitly based on SCT [45]. When using theory it is particularly helpful to measure and document changes in behavioral constructs or antecedents of behavior the theory has reified. Among the thirteen studies based on some theory, four did not measure any antecedent of behavior change. For studies that did, self-efficacy was the most commonly measured antecedent. This again was not surprising, since self-efficacy is the principle construct of SCT. From this review it can be concluded that there is an apparent need in this area. More research is needed in the advancements of operationalizing theoretical constructs into programmatic activities, and research is needed in evaluating what programmatic activities are ultimately most beneficial for behavior change. For example self-efficacy has been found to be significantly associated with exercising daily for 30 minutes and consuming five servings of fruits and vegetables among fifth grade children [46]. While future interventions should target self-efficacy for both behaviors, program activities may not be the same, given the inherent differences in the two behaviors. Along side this recommendation, the need to validate instruments measuring behavioral antecedents is greatly needed. Smith [47] found that among all articles published from 2006−2007 in four of the top journals in Public Health Education (Health Education and Behavior, Health Education Journal, Health Education Research, and International Electronic Journal of Health Education), less than half (41.6%) reported any psychometric property when needed, and the most commonly reported coefficient was Cronbach’s alpha. For step-by-step guidance on the proper methodologies for validating surveys measuring theoretical constructs, please refer to Barry and colleagues [48]. 

With regards to the duration of the interventions in this review, they greatly varied from 3 weeks to 3 years. Since there is no universally accepted criterion for what is considered a ‘brief’ or ‘long term’ intervention, it was difficult to fully describe this feature in this review. However, by using the criteria Cook-Cottone and collegues [10] used in their meta-analysis of school-based obesity prevention interventions (programs ranging from 0 to 12 weeks were considered short, 13 to 27 weeks as low-moderate, 28 to 32 weeks as moderate, and those lasting more than 32 weeks long) it was found that a majority (10 interventions) could be considered *short*, 5 were *low-moderate*, and 5 were *long*. From these findings it appears that greater efforts have been given to shorter interventions, which may have contributed to the low amount of impact variables found to be significantly mediated for the studies in this review. In the future longer interventions (greater than 12 weeks) should be developed and evaluated to contribute to the existing evidence.

Table 2 presents various methodological issues for the studies in this review. The first issue is in regards to the impact measures. The most commonly reported measure was some type of weight status, body composition or other functional assessment (*n* = 19 or 76% of studies) following behavioral measures (*n* = 17 or 68% of studies) and the least common measure used were behavioral antecedents (*n* = 13 or 52% of studies). Very few studies (*n* = 4 or 16% of studies) included all three types of measures, and most studies used at least two types (*n* = 16 or 64% of studies). There were five studies (or 20% of studies) that only included one type of measure. To evaluate physical activity and diet a variety of methods were used. Both behaviors can be measured using either subjective (or self-report) or objective (or independently measured) means. Physical activity measurements mainly relied on self-report, as four studies utilized brief surveys [18,19,20,21,22], three utilized physical activity recalls [23,25,26], and three [35,38,39] used accelerometry. One study also used parents to recall the amount of physical activity their child(ren) participated in over a period of time [40]. Diet was similar as six studies relied on self-report [21,23,30,33,36,38,39,41], and three relied on parent recalls [29,34,40]. Self-report methods did vary however, with some studies utilizing surveys and others using 24-hour recalls. Planning models such as the Precede-Proceed model call for a comprehensive evaluation of interventions, and often stress the importance of evaluating all three types of measures. By including all three, researchers can also better understanding whether or not program activities are robust enough to impact behavioral antecedents, whether the impact on the behavioral antecedents are sufficient for mediating behavior change, and finally whether behavioral changes are strong enough to impact other variables such as weight status or body composition. Future studies would benefit from including all three types of measures described in this review.

With regards to sample size, seven studies reported an *a priori* sample size calculation, 5 of which were RCT’s, and two had a quasi-experimental design. As Eng [49] reported, it is important for studies to have an adequate sample size, since it directly impacts the statistical power of the study. Studies with inadequate power run the risk of reporting false-negative findings, which are commonly known as a type II error. This is positive finding, that most researchers evaluating RCT’s are recruiting an adequate number of research participants. Most of the quasi-experimental studies did not have sample size calculations, however this could strengthen their results. Sample size calculations are not generally warranted for pilot studies, since their true purpose is to test the feasibility of the intervention, and gather information to justify future implementation. Future studies should continue reporting their *a priori* sample size calculations, especially for RCT’s.

The next issue reviewed in this article deals with the utilization of some type of process evaluation. Monitoring the implementation of obesity prevention interventions, or any type of health promoting program, is extremely important. This is especially true when multiple facilitators implement interventions across multiple sites for the same study. By failing to monitor program activities, researchers run the risk of making what is known as a type III error, where weak or null results can be attributed to poorly executed or incorrectly implemented interventions [50]. Most process evaluations focus on two dimensions; dose, or the amount of time research participants spend engaged in program activities, and fidelity, or to what extent an intervention was delivered according to the intended delivery [50]. While few frameworks exist for process evaluations, Saunders and colleagues [51] outline a useful six-step framework for developing and using six types of process evaluations for health promotion programs. The steps include: fidelity (whether the intervention was implemented as planned), dose delivered (assurance that program lessons were implemented in order and for the amount of time planned), dose received (whether the intervention was well received by the participants), reach (attendance), recruitment (an assessment of what tasks were implemented to approach and invite participants to be involved with the study), and context (aspects of the environment that could have influenced the implementation of an intervention or study variables or contamination the comparison group might have by being exposed to the experimental program). From the studies in this review, 19 (or 76% of studies) reported using at least one type of process evaluation. In a further evaluation of these studies, it was found that attendance (or *reach)* was the most commonly used process evaluation method. More attention should be given to process evaluations in future studies, and researchers should consider using the Sauders model [51], or other models such as the Process Evaluation Model (PEM) [52], or the RE-AIM Framework, which stands for Reach, Effectiveness, Adoption, Implementation, and Maintenance [53].

Another common limitation in the design of the studies in this review was that only three studies evaluated any measure past the time of post intervention. Follow-up evaluations are greatly needed with obesity prevention research, to show whether effects are sustained after a set amount of non-intervention time. This is especially true for measures of weight status, such as BMI-percentile or *z*-scores; while weight status may not change in the short-term, there is a great deal of interest in showing longer-term weight maintenance of children participating in experimental interventions. Drawing upon Prochaska’s Transtheoretical Model, six months appears to be an appropriate amount of time to implement a follow-up, since the theory purports that individuals typically need at least this amount of time to maintain a behavior change [54]. Nonetheless, while a six month follow-up would be beneficial, practically any follow-up assessment would be beneficial for evaluating a program’s ability to make long-lasting behavior change.

A final issue not appearing on Table 2 is with regards to reporting the use of intra-class correlation (ICC) in data analysis, when appropriate. While RCT’s do appear to be the strongest design for evaluating obesity prevention programs, researchers can rarely assign children to intervention conditions and often must assign groups of children to conditions, such as children attending the same school or after school program. Stevens and colleagues [55] explain that RCT’s carry the unique challenge of having correlations among study variables within these assigned groups. The magnitude of this association is known as the ICC. It is important to be aware that ICC can impact study outcomes and should be properly controlled for, however is not always properly used or recognized in the literature. In a review of 59 grouped RCT’s authors concluded that only 54% used “appropriate analyses” accounting for ICC, while 25% used a mixture of ‘appropriate and inappropriate analyses’, and 20% used ‘all inappropriate analyses’ not accounting for ICC [56]. The magnitude of this correlation has the potential to impact study results, which could lead to misleading or erroneous conclusions. In the articles reviewed for this study five of the seven RCT’s mentioned using the ICC as part of their data analysis. As more rigorous studies are employed in this area, future researchers should be sure to take the ICC into account in data analysis. 

## 5. Implications for Future Studies

There has been great interest in the area of obesity prevention in the after school setting, and further work in evaluating and implementing these types of interventions is greatly needed. The following outlines implications and methodological recommendation for future studies. First, obesity prevention interventions should target both physical activity and nutrition behaviors. Increasing physical activity was a common theme among many interventions in this review, but reducing sedentary activities was not as heavily targeted. Sedentary activities, such as time spent watching TV or on the Internet, is an important modifiable behavior, as it has been shown to be an independent risk factor for overweight and obesity [57], as well as metabolic risk, including blood pressure and hypercholesterolemia [58]. Important dietary behaviors to target includes those outlined by the 2005 American Medical Association’s expert committee for recommendations regarding the prevention, assessment, and treatment of child and adolescent overweight and obesity, which included; fruit and vegetable consumption, eating breakfast, eating out at restaurants (particularly fast food restaurants), eating family meals, consuming sugar-sweetened beverages and water, consuming calorically dense/nutrient poor foods, and limiting portion sizes [59]. 

There is also need for interventions to be based on behavioral theories. As researchers and practitioners, we must remember that interventions do not intrinsically modify behaviors; rather program activities we employ target behavioral antecedents, which in turn are theorized to impact behaviors. Therefore, interventions must clearly operationalize and measure these constructs, which will result in stronger evidence for confirming or rejecting the utility of a given theory for a specified behavior, among a target group. These instruments must also be psychometrically tested to establish they are valid and reliable measures, since measuring behavioral constructs are exclusively done by self-report. 

Finally, researchers and health educators should greatly consider implementing more than one type of process evaluation, as they are the only means to assure a given program was delivered with fidelity. While it is understood that this requires additional time during the stage of program planning, there are simple and inexpensive means of using process evaluations that would not require additional personnel support or time from the program facilitator. For example, the program facilitator can complete a self-check after each lesson to document the completion of program activities, assure each lesson is implemented for the amount of time planned by using a stop watch, take attendance at each lesson, and assure each lesson is implemented in the order originally prescribed. These recommendations and the others covered in this article will advance our knowledge in this area to better evaluate the effectiveness of this intervention strategy, and give guidance for future programming in the after school environment. 
